# Polytetrafluoroethylene Films in Rigid Polyurethane Foams’ Dielectric Permittivity Measurements with a One-Side Access Capacitive Sensor

**DOI:** 10.3390/polym13071173

**Published:** 2021-04-06

**Authors:** Ilze Beverte, Ugis Cabulis, Sergejs Gaidukovs

**Affiliations:** 1 Institute for Mechanics of Materials, University of Latvia, Aizkraukles street 23, LV-1006 Riga, Latvia; 2 Latvian State Institute of Wood Chemistry, Dzerbenes street 27, LV-1006 Riga, Latvia; cabulis@edi.lv; 3 Faculty of Materials Science and Applied Chemistry, Institute of Polymer Materials, Riga Technical University, P.Valdena street 3/7, LV-1048 Riga, Latvia; Sergejs.Gaidukovs@rtu.lv

**Keywords:** polyurethane foams, dielectric permittivity, capacitive sensor, one-side access, adverse effects, protection, PTFE films

## Abstract

As a non-metallic composite material, widely applied in industry, rigid polyurethane (PUR) foams require knowledge of their dielectric properties. In experimental determination of PUR foams’ dielectric properties protection of one-side capacitive sensor’s active area from adverse effects caused by the PUR foams’ test objects has to be ensured. In the given study, the impact of polytetrafluoroethylene (PTFE) films, thickness 0.20 mm and 0.04 mm, in covering or simulated coating the active area of one-side access capacitive sensor’ electrodes on the experimentally determined true dielectric permittivity spectra of rigid PUR foams is estimated. Penetration depth of the low frequency excitation field into PTFE and PUR foams is determined experimentally. Experiments are made in order to evaluate the difference between measurements on single PUR foams’ samples and on complex samples “PUR foams + PTFE film” with two calibration modes. A modification factor and a small modification criterion are defined and values of modifications are estimated in numerical calculations. Conclusions about possible practical applications of PTFE films in dielectric permittivity measurements of rigid PUR foams with one-side access capacitive sensor are made.

## 1. Introduction

Rigid polyurethane foams (PUR foams) exhibit a low dielectric interference, their permittivity is nearly non-dispersive therefore a good dielectric performance can be ensured in a wide frequency range. That makes PUR foams an appropriate material for components of mechanical engineering, machine building, aerospace vehicles, electronics’ packaging, electric insulators, radomes—the shielding structures for out-door telescopes, locators, antennas, etc., providing a radio-frequency transparent layer along with dimensional stability [1,2,3].

As a non-metallic composite material, widely applied in industry, PUR foams require knowledge of their dielectric properties. Several values of dielectric permittivity (permittivity) are reported in an experimental investigation of rigid PUR foams with a capacitor at frequencies 100 Hz and 10 MHz according to ASTM D1673 [4]. In [5] a one-side access (OSA) capacitive sensor was used to determine permittivity at low frequencies 10 Hz–0.33 MHz for (1) petrochemically-rigid PUR foams, density 32–539 kg/m^3^: 1.059 ≤ ε ≤ 2.067 and for (2) monolithic petrochemical-origin polyurethane, density 1280 kg/m^3^: 3.47 ≥ ε ≥ 3.31. To characterise the dielectric dispersion, the dropping factor was introduced and calculated for the investigated PUR foams of densities up to 400–450 kg/m^3^ as F ≤ 5.0% and F ≈ 6.0% for monolithic petrochemical polyurethane.

Experimental determination of rigid PUR foams dielectric properties with capacitive sensors meats several problems. PUR foams release gasses from foaming process [1,2] that may cause oxidation or other chemical processes in the metal of the capacitive sensor’s active area (e.g., brass or copper). PUR foams can carry considerable static electricity (caused by exploitation and processing conditions) that damages the electronic system of sensors [5]. The daylight degraded and mechanically-processed surfaces of PUR foams’ drop plastic micro-particles [1,2], leading to mechanical contamination of the sensor’s active area, thus the accuracy of the permittivity measurements is reduced.

Protection of the capacitive sensor’s active area from adverse side effects caused by the PUR foams’ test objects has to be ensured. Different solutions for protection of capacitive sensor’s electrodes exist in experimental investigation of other dielectric materials. In [6] the copper plates of the electrodes are covered with a protecting varnish to avoid the oxidation from contact with the test object (The soil) and to eliminate effect of electric load conduction through the dielectric. A protective layer is often placed over the electrodes in a capacitance sensor to prevent the direct contact to the test object [7]. The issues in sensor construction include the choice of materials for the electrodes, insulation layer, substrate etc. When the active area of sensor’s electrodes is coated with a protective layer, the coating has to satisfy certain requirements. The permittivity value for the material of protective layer should be chosen close to the value of material of the test object to reduce modification of the electric field [8]. The thicknesses of the protective layer can influence the signal strength and the sensitivity distribution therefore it has to be optimised [9]. The material of the layer has to have a nearly constant real part of the complex permittivity with respect to the frequency of electric field (non-depressiveness) and small imaginary part of the same not to cause dielectric losses. Since the test object comes into a direct contact with the sensor’s surface, the protective layer has to withstand certain mechanical wear as well. An analysis of scientific information sources revealed a lack of investigations and reliable experimental data on OSA capacitive sensor’s protective coverage and coating in permittivity measurements of cellular plastics, including rigid PUR foams. The major technical difficulties, connected to the plastics foams themselves, lie in extremely low permittivity values; e.g., rigid PUR foams of density 31 kg/m^3^ exhibit permittivity ≈ 1.065 (1 kHz), that is only ≈ 6% higher than the permittivity of vacuum [5].

When looking for a high-performance coating that offers exceptional temperature, chemical, friction, and abrasion resistance, the fluoropolymer coatings are often the solution. A fluoropolymer coating application is a generic term for xylan or polytetrafluoroethylene (PTFE) coatings. PTFE is a semi-crystalline non-polar polymer with the main chain [CF_2_-CF_2_]_n_; the brand name of PTFE-based formulas is “Teflon”. PTFE is reported to have frequency practically non-dependent permittivity ε and low dissipation factor tgδ. Those features of PTFE resins are also relatively independent of fabrication conditions. In [10] PTFE is reported to exhibit absence of significant dielectric losses up to its first order transition point at temperature T = 327 °C. At T = 23 °C and frequencies f = 10^2^, 10^3^, 10^4^, and 10^5^ Hz: ε′ = 2.001–2.002 and tgδ = 1–2 × 10^−4^. In [11] the low loss tangent of PTFE is explained as a consequence of the symmetrical conformation of the polymer backbone, which neutralizes the dipole forces of the C–F bonds yielding a net zero dipole moment. At 1 MHz dielectric constant of PTFE is determined as 2.1 and dissipation factor tgδ < 0.0002. In [12] a wide range of experimental data of PTFE investigations is reported. At room temperature T = 300 K, at frequencies 75 Hz and 500 Hz: tgδ < 1 × 10^−4^, at 1 kHz: tgδ < 1–6 × 10^−4^. At 5 kHz–2.2 MHz: tgδ < 1 × 10^−4^, at 1 kHz–316,000 Hz: tgδ = 2–6 × 10^−4^, 1 kHz–100 kHz: tgδ = 1.5–5.5 × 10^−4^. Dielectric permittivity at 100 Hz–1 MHz is given as ε′ = 2.2–2.0. Matis [13] reports values of dielectric permittivity and loss tangent of PTFE at two frequencies f = 100 Hz and 1 MHz: ε = 2.1 = const. and tgδ = 0.0005 and 0.0002. The lack of dependence of ε and tgδ on frequency is explained by PTFE having non-polar molecules and exhibiting only elastic polarization. Naidu [14] gives the following values at frequencies f = 50 Hz–1 MHz: ε = 2.3–2.8 and tgδ < 0.0002. Askeland [15] provides the data at 60 Hz–1 MHz: ε = 2.1 and 2.1 and tgδ = 0.00007 at 1 MHz. Jiang [16] reports dielectric constant of PTFE at 1 MHz as ε = 2.2 and dissipation factor tgδ < 0.00012.

The unique dielectric characteristics permit to use PTFE as thin wall insulation, as jacketing for computer wires and special control wires, for tubing and sleeving of capacitors, resistors, terminal junctions and solder sleeves. Due to small modifications caused by PTFE in the measured values of the dielectric properties of sample materials, PTFE spacers are used to adjust the gaps between electrodes in liquid parallel plate sample cells in the *BDS*-1308 broadband dielectric spectrometer (Novocontrol Technologies GmbH and Company KG (Montabaur, Germany)). Advanced technologies as a liquid spray or as a powder coating are developed for coating PTFE on metals, including brass that is often used for sensor’s electrodes.

The value of PTFE’s permittivity ε ≈ 2.1 coincides with that of rigid PUR foams of density 600 kg/m^3^ that corresponds to the middle of PUR foams’ full range of densities: at 31 kg/m^3^ permittivity ≈ 1.065; at 550 kg/m^3^ ε ≈ 2.1 and for monolithic polyurethane, density 1280 kg/m^3^, permittivity ≈ 3.3 (1 kHz) [5]. PTFE is nonreactive, it reduces mechanical friction and wear (PTFE’s static frictional coefficient ≈ 0.04), therefore, a longer service time of the protective coating can be expected. The dielectric and mechanical properties suggest PTFE’s usage in protection of OSA capacitive sensor’s active area in PUR foams’ permittivity measurements.

For OSA capacitive sensors the intensity maximum of low-frequency excitation field is situated in the direct vicinity to the active area of the electrodes [17]. Penetration depth of excitation field into the test object determines its thickness appropriate for permittivity measurements. Different criteria have been proposed for definition of penetration depth. In [18] the control depth of an OSA capacitive sensor is defined as distance to a conductive plane, parallel to the working surfaces of electrodes, placed in air, at which the capacitance, implemented by the conductive plane equals to a certain, predetermined quantity from the working capacitance of the capacitor. In [19] the overall capacitance of the system of two OSA sensors is measured and a half of the smallest distance between the planes of electrodes of the two sensors, starting from which the overall capacitance of the system remains constant, is taken as the depth of control zone of the OSA capacitive sensor. In [20] penetration depth of a concentric coplanar capacitive sensor is defined by identifying the test object thickness for which the capacitance is 10% smaller than its value when in contact with a similar, but infinitely thick, test-piece. When this condition is satisfied, the sensor penetration depth is equal to the test object’s thickness and is dependent on the permittivity of the test object. To determine thickness of PUR foams’ test object, appropriate for accurate permittivity measurements, penetration depth of excitation field into PTFE and rigid PUR foams has to be investigated.

In the given interdisciplinary research impact of PTFE films, thickness 0.20 mm and 0.04 mm, in covering or simulated coating of the active area of OSA capacitive sensor’ electrodes on the true dielectric permittivity spectra of rigid petrochemical- and bio-origin PUR foams is estimated by means of (1) a modification factor and (2) a small modification criterion. Single and complex samples, comprising PUR foams and PTFE films, are investigated experimentally at two calibration modes. Numerical estimations of the modifications in the true permittivity spectra are acquired for both PTFE films, thickness 0.20 mm and 0.04 mm, covering or coating the active area of OSA capacitive sensor’ electrodes. Conclusions about possible applications of PTFE film, thickness 0.04 mm, in protection of one-side capacitive sensor’s active area from adverse effects caused by the PUR foams’ test objects during dielectric permittivity measurements are made.

## 2. Materials and Methods

### 2.1. PUR Materials

Rigid closed-cell petrochemical PUR foams were made in laboratory conditions in a range of apparent core density 80 kg/m^3^ ≤ ρ ≤ 850 kg/m^3^, in blocks, in (a) open, free-rise moulds (25 cm × 25 cm × 20 cm) and (b) closed cylindrical polypropylene moulds (height = 8.0 cm and inner diameter = 10.0 cm) according to the technology and formulations given in [5]. Responding to the needs of bioeconomy, biopolyol was synthesized from Latvia-grown rapeseed oil by the trans-esterification method with triethanolamine (molar ratios 1 M:2.5 M and 1 M:2.9 M), in an environmentally friendly process, without emission of harmful substances, at temperatures T = 175 °C ± 5 °C and rigid closed-cell polyurethane biofoams were made in a range of apparent core density 80 kg/m^3^ ≤ ρ ≤ 450 kg/m^3^. Apparent core density of PUR foams (density) were determined according to ISO 845:2006. Differences in densities were achieved by varying the amount of physical or chemical blowing agents.

To make monolithic petrochemical polyurethane, a liquid mixture of the same formulation as for PUR foams was poured in polyethylene ampoules with an inner diameter 25.4 mm and length of 115 mm; no foaming agent was added. The ampoules were centrifuged for ~20 min at 5000 rpm in a centrifuge Sigma 3-30KS (Sigma Laborzentrifugen GmbH (Osterode am Harz, Germany)) to eliminate air inclusions created by mechanical mixing [5]. The PUR rods were removed from ampoules and samples were made. The developed technology ensured ≈ 2/3 of the length of cylindrical part free from gaseous inclusions.

Industrially manufactured rigid petrochemical PUR foams from a European producer Sika JSC (Baar, Switzerland) and General Plastics Manufacturing Company, (Tacoma, USA), were investigated as well to compare the results of the four groups of PUR foams.

### 2.2. Test Objects and Experiments

**Figure 1:**
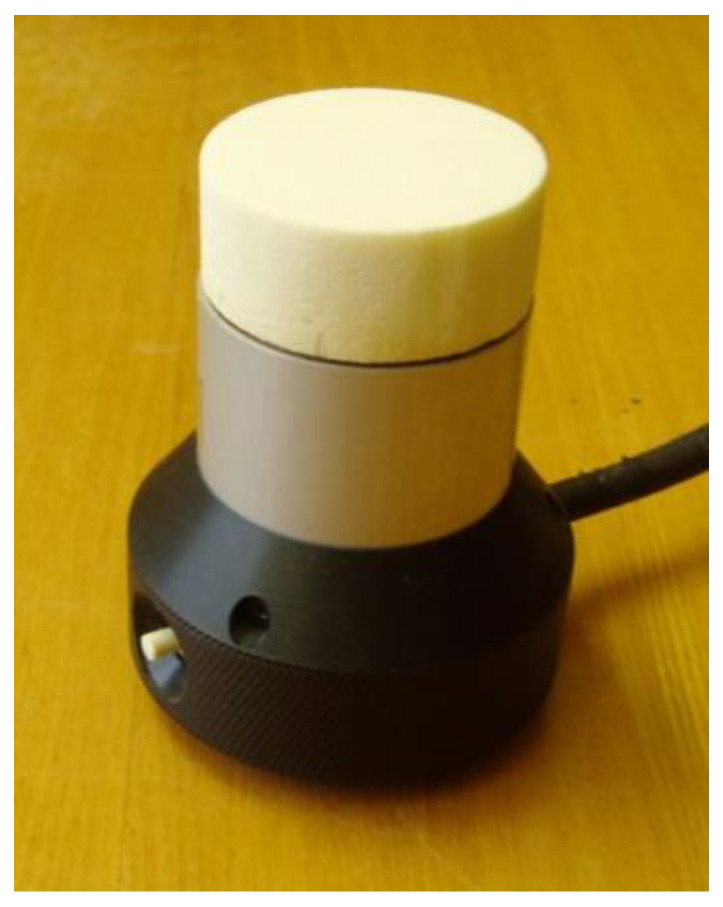
OSA sensor and a PUR foams’ sample.

**Figure 2:**
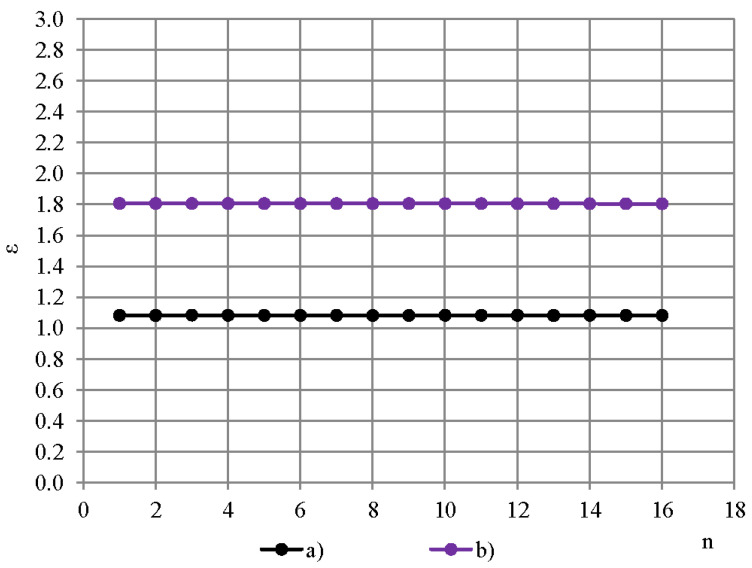
Measured permittivity spectra of a PTFE film, 0.20 mm: (a) OSA sensor and (b) BDS-50.

Permittivity was measured with an innovative, experimental dielectric spectrometer equipped with a capacitive sensor of OSA type [21]. Stray-immune capacitance measurements were carried out by using active guarding of the sensing electrode to increase the sensitivity and accuracy of the measurements. The test object was placed on the active area of the OSA capacitive sensor (OSA sensor), the diameter of the annular outer electrode D_0_ = 43 mm, Figure 1, and was excited via electrodes, by an electrical field generated by sinusoidal voltage signals. The amplitude value of the sinusoidal excitation signals U_0_ = 20 V. The signals were generated at discrete frequencies, increasing in a geometric progression:

f_n_ = f_1_, 2f_1_, ..., 2^(n − 1)^f_1_ Hz, where f_1_ = 10 Hz, n = 1, 2, ..., 16; f = 10, 20, …, 327,680 Hz,(1)

where n is the ordeal number of frequency. The accuracy of the dielectric spectrometer in permittivity measurements, in conditions of repeatability, was estimated with the expanded uncertainty U95.45% = 0.01. The following test objects were investigated: (1) single samples of PUR foams and monolithic polyurethane, diameter D = 45 mm, thickness h = 20–25 mm, Figure 1, and (2) complex samples “A single PUR foams’ sample + PTFE film”. The true dielectric permittivity spectra ε_t_ were measured on single samples, when calibration of the spectrometer was made before the measurement series, with regard to the measurement value, delivered by the OSA sensor in air [21].

The true permittivity spectra, measured with the OSA capacitive sensor were compared to those measured with a Broadband Dielectric Spectrometer BDS-50, comprising a parallel plate capacitor, supplier: Novocontrol Technologies GmbH and Company KG (Montabaur, Germany), operating frequencies: 3 μHz–40 MHz, samples: discs of diameter 20–21 mm or 30–31 mm and thickness 0.1–2.0 mm. PUR foams in density range 95–1280 kg/m^3^ were tested. The relative difference between data provided by the two apparatuses remains ≤4% in the entire frequency range.

The measured value of dielectric permittivity was determined in two kinds of experiments: (1) for complex samples “A single PUR foams’ sample + PTFE film”, when calibration was made before the measurement series, with regard to the measurement value, delivered by the OSA sensor in air and (2) for complex samples “A single PUR foams’ sample + PTFE film”, when calibration was made before the corresponding measurement series, with regard to the measurement value, delivered by the OSA sensor in air, but covered with a PTFE film. The impact of PTFE films of thickness 0.20 mm and 0.04 mm was investigated.

In experiment no. 1, the OSA sensor’s active area is *covered* with a protective PTFE film *temporarily* during the measurement, and the film is removed during calibration. It gives the measured value of permittivity of a complex sample “A single PUR foams’ sample + PTFE film”. The value is denoted as the measured value of permittivity of a PUR foams’ sample ε_T_. Experiment no. 2 simulates the case when OSA sensor’s active area is *coated* with a protective PTFE film *permanently*. It gives the measured value of permittivity of a complex sample “A single PUR foams’ sample + PTFE film”, when the measurement as well as calibration have to be made with a PTFE film on the OSA sensor. The value is denoted as the measured value of permittivity of a PUR foams’ sample ε_T′_.

For measurements with PTFE films, a film element, length ≈ 30 cm and width ≈ 6 cm, was fixed in the horizontal plane between two massive dielectric parallelepiped posts at each side. The OSA sensor was placed beneath the film, the film was stretched to ensure a smooth adjoining to the OSA sensor’s active area and the measurement was made. Then a complex sample was formed: A single sample was put on the film and the measurement was made.

Due to limited transversal and lateral dimensions of the lab-made monolithic polyurethane (Rods of diameter ≈ 25 mm, length of the cylindrical part ≈ 60 mm), permittivity of monolithic polyurethane was measured on two semi-cylinders, cut from a polyurethane rod, ρ = 1280 kg/m^3^. Side-by-side the two semi-cylinders form a round cylinder, thickness h = 12 mm, diameter D = 45 mm, fully covering the active area of the OSA sensor. It was proved in [5] that the relative error between the values of permittivity of a cylindrical sample and two semi-cylinders does not exceed 0.5%.

A density gradient may exist in the moulded PUR foams’ blocks along rise direction, especially in a free rise [1,2]. To reduce its impact, samples were picked from the most homogeneous parts of the blocks. Air gaps between the active surface of the sensor and the sample may reduce the measured value of permittivity [3,13,17], therefore, surfaces of the samples were processed to ensure an appropriate degree of smoothness. Triboelectricity that accumulated on the samples at grinding was channelled away by placing samples on a conductive, grounded surface. Prior to measurements the samples were conditioned by storing at temperature T = 21 °C ± 1 °C and relative humidity RH = 45% ± 5% for a minimum of 24 h. Three successive measurements were made for each permittivity data point.

### 2.3. Dielectric Losses

The dielectric loss part ε′′(f) of the complex permittivity $\tilde{\varepsilon }$(jf) = ε′(f) − jε′′(f) was evaluated for PUR foams and bulk PTFE, based on data from direct experiments as well as scientific information sources. Dielectric losses of PUR foams were measured on Broadband Dielectric Spectrometer BDS-50 (Novocontrol Technologies GmbH and Company KG (Montabaur, Germany)), comprising a parallel plate capacitor, on samples of thickness 2 mm and diameter 30 mm. Five measurements were made for each data point. For lab-made PUR foams of densities 95–222 kg/m^3^ dielectric losses were measured as ε′′ = 0.0022–0.0063 at 1 kHz and ε′′ = 0.0032–0.0084 at 0.1 MHz. For monolithic lab-made polyurethane ε′′ = 0.042 at 1 kHz and ε′′ = 0.088 at 0.1 MHz. The acquired values are in a good correspondence with the experimental data reported in [22].

Dissipation factor for bulk PTFE at frequencies f = 50 Hz–1 MHz, at room temperature, is reported in [13,14,15,16] as tgδ < 0.0002. Taking into account ε′′ = ε′tgδ, ε′′ < 0.00042. BDS-50 gave the following results at 1280 Hz–81,920 Hz ε′′ = 0.00026–0.00027. For both materials the loss part is small in the considered frequency range, $\tilde{\varepsilon }$(jf) ≈ ε′(f) and ε′(f) = ε(f) is further referred to as permittivity.

### 2.4. Penetration Depth

Intensity maximum of the dielectric spectrometer’s low-frequency excitation field is situated in the direct vicinity to the active area of the OSA capacitive sensor’s electrodes [17]. Figure 2 gives the measured permittivity spectra of a PTFE film, thickness 0.20 mm, determined with (a) dielectric spectrometer equipped with the OSA sensor and (b) BDS-50. The homogeneous electric field of BDS-50 provides a spectrum of the measured permittivity values closer to those of the bulk PTFE ε ≈ 2.1 than the inhomogeneous and fast-attenuating electric field of the OSA sensor.

**Figure 3:**
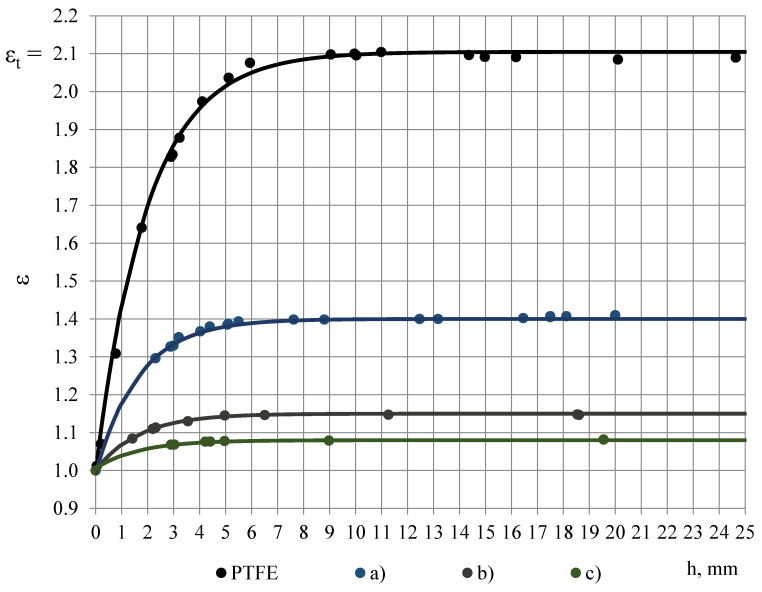
Measured value of permittivity in dependence of sample’s thickness: PTFE and lab-made PUR foams, density (a) 228, (b) 88 and (c) 50 kg/m^3^ (experimental data and model functions, 1 kHz).

Penetration depth of the excitation field is a crucial characteristic for a OSA capacitive sensor in determination of appropriate thickness of a single sample, depending on the parameters of the sensor and dielectric material of the sample [17,20]. To estimate penetration depth into PUR foams and PTFE films, first the measured value of permittivity of PTFE in dependence of thickness of cylindrical samples, diameter D_0_ ≈ 45 mm, thickness h = 0.04–24.6 mm, was determined experimentally with the OSA sensor, Figure 3. Layers were cut from the top of ~ 25 mm thick PTFE sample; permittivity of the remaining sample was measured and plotted against its thickness. Three measurements were made for each data point. Average density of the samples was calculated as 2177 ± 38 kg/m^3^ (±1.8%) that corresponds with the density values, reported in [12].

For thickness 11 mm ≤ h ≤ 25 mm the average permittivity was calculated 2.10 ± 0.011 (the expanded uncertainty U95%), at 1 kHz, and it is considered as the true value of PTFE’s permittivity ε_t_, not depending on thickness. The value ε_t_ = 2.10 is in a good correspondence with the values reported in [13,15,16]. The experimental data is modelled with an exponential function, Figure 3:

ε = ε_t_ − e^(0.1 − 0.5 h)^. (2)

At other frequencies f_1_, f_2_, …, f_16_ the relationship ε = ε(h) remains practically the same due to insignificant dispersion of PTFE’s permittivity. 

The measured value of permittivity in dependence of sample’s thickness was determined for the lab-made PUR foams in the same way, modelling the experimental data with the exponential function ε = ε_t_ − ^(A+Bh)^, where ε_t_—the true value of PUR foams permittivity, A and B—numerical coefficients, depending on the PUR foams’ density. To define penetration depth, the measured value of electric susceptibility is considered:

χ = ε − 1.00.(3)

It permits defining the penetration depth for extremely light-weight PUR foams having permittivity value, close to that of vacuum; for any reduction percentage, e.g. if, in order to determine the penetration depth, a criterion of a 10% reduction of the true permittivity [20] is applied directly to the true permittivity of rigid PUR foams of density 50 kg/m^3^ (ε_t_ = 1.08 at 1 kHz), then:

ε_10%_ = ε_t_ − 10% ε_t_ = 0.97 < **ε**_0_; (4)

where ε_0_ = 1.00 is permittivity of vacuum. That does not correspond to the definition of the true permittivity as a quantity always having values ≥ε_0_. Applying the mentioned criterion to the electric susceptibility solves the contradiction. In the given investigation the penetration depth of electric field is defined as the thickness h_3%_ of a sample, at which the measured value of electric susceptibility χ is 3% less than the true value of electric susceptibility χ_t_ of an infinitely thick sample. Then for PTFE:

χ = [χ_t_ − e^(0.1–0.5 h)^ − 1.0 and χ_3%_ = χ_t_ − 0.03χ_t_ = 0.97χ_t_ = 1.07. (5)

Then penetration depth h_3%_ for PTFE is calculated as:

h = −2.0 [ln(χ_t_ − χ(h)) − 0.1]; h_3%_ = −2.0 [ln(χ_t_ − χ_3%_) − 0.1] = 7.0 mm. (6)

Thickness of the PTFE films 0.20 and 0.04 mm << h_3%_, hence it can be concluded that the electric field, generated by sinusoidal voltage signal at frequencies f_1_, f_2_, …, f_16_ fully penetrates the PTFE films and reaches PUR foams’ sample. 

For PUR foams of densities 50–228 kg/m^3^ and ε_t_ = 1.14–1.42 (1 kHz), penetration depth was determined as 5.72 mm ≤ h_3%_ ≤ 5.87 mm ± 0.02 mm. That corresponds to the conclusions in [20]: In the meaning of the given penetration depth definition, penetration depth of a concentric coplanar capacitive OSA sensor increases as the permittivity of the sample increases. In order to achieve the same 3% difference, samples with high ε_t_ values need to have bigger penetration depth h_3%_, whereas samples with low ε_t_ values can have smaller h_3%_ to achieve the same percentage of difference [20]. The PUR foams’ single samples have to be thick enough to provide the true value of permittivity, therefore, 3–4 times higher thickness than the penetration depth h_3%_ was taken as appropriate for PUR foams’ samples of densities 50–1280 kg/m^3^: h ≈ 20–25 mm.

### 2.5. Dropping Factor

**Figure 4:**
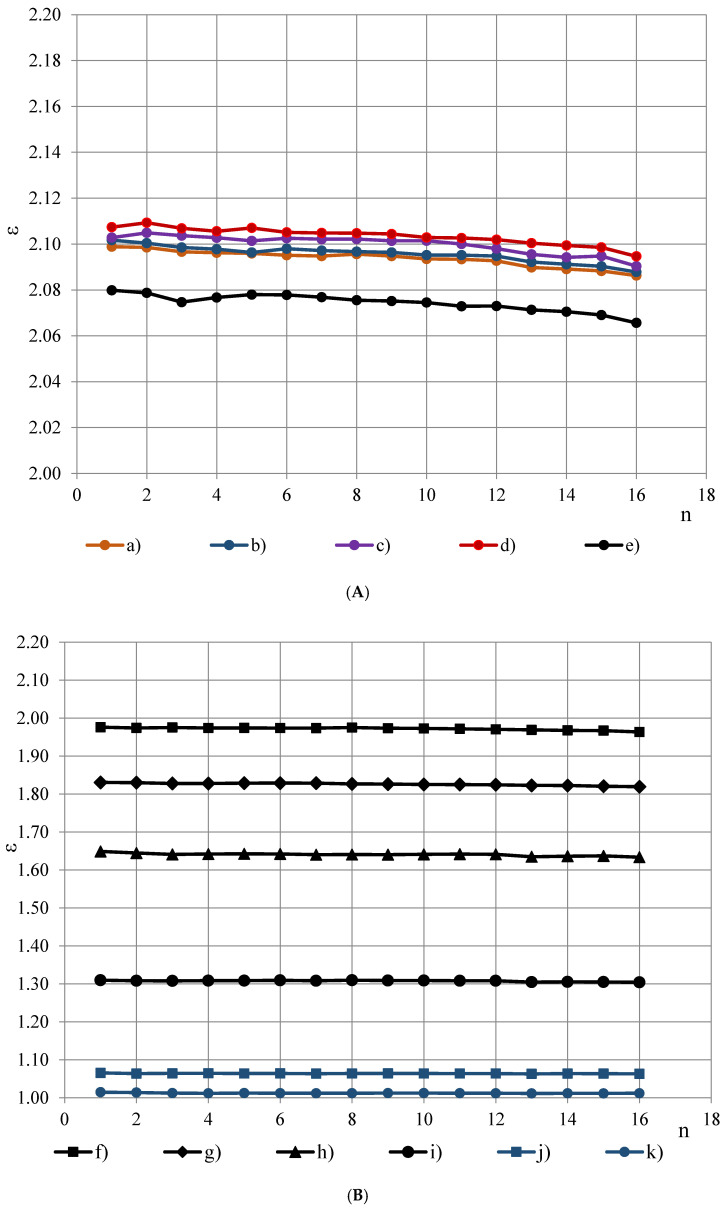
(**A**) Permittivity spectra of PTFE samples of thickness (a) 24.64, (b) 20.10, (c) 16.18, (d) 10.99 and (e) 5.94 mm. (**B**) Permittivity spectra of PTFE samples of thickness (f) 4.10, (g) 2.90, (h) 1.77 mm, (i) 0.77 and PTFE films of thickness (j) 0.20 mm and (k) 0.04 mm.

Dielectric dispersion is the dependence of permittivity of a dielectric material on the frequency of an applied electric field. Because there is a lag between changes in the external electric field and changes in polarization in a dielectric, the permittivity of a dielectric is a complicated function of frequency of the electric field [3]. Dielectric dispersion of both components of the complex sample (PTFE and PUR foams) has to be evaluated. PTFE is known to have a small frequency dependence of permittivity in frequency range 100 Hz–10 MHz [10,11,12,13,14,15,16]. To evaluate frequency dependence of PTFE’s permittivity in the frequency band f = 10, 20, …, 327,680 Hz, permittivity spectra of PTFE samples of different thickness h were measured with the OSA sensor, Figure 4A,B. Three repeated measurements were made for each sample.

**Figure 5:**
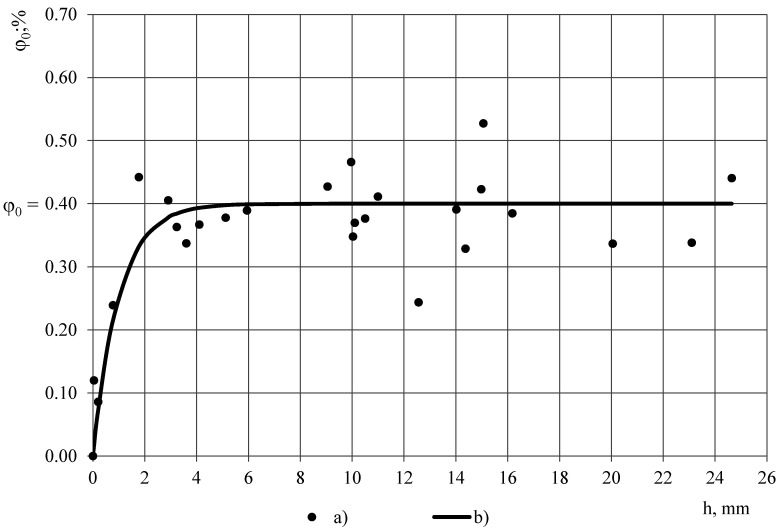
Dropping in dependence of PTFE sample’s thickness: (a) Experimental data and (b) model function.

The spectra ε(f_n_) were approximated with 2-nd order polynomials, e.g., for the PTFE films, Figure 4B, (j) and (k):

ε = 0.000003n^2^ − 0.000136n + 1.064500 and ε = 0.000017n^2^ − 0.000411n + 1.013888, (7)

where the ordinal number n of the frequency is calculated from Equation (1) as n = (logf_n_ − 1)/log2 + 1. To characterise the dielectric dispersion, the dropping of permittivity φ was calculated from the most monotonous part f_3_, f_4_, …, f_14_ of the approximated spectra as:

φ = [ε(f_3_) − ε(f_14_)]/ε(f_3_), (8)

where ε(f_3_) and ε(f_14_)—permittivity at f_3_ = 40 Hz and f_14_ = 81 920 Hz. For PTFE films φ ≈ 0.09% at thickness h = 0.20 mm and φ ≈ 0.12% at h = 0.04 mm, Figure 5. Starting from a certain value of h ≈ 5 mm, dropping becomes independent of thickness. The thickness-independent value of dropping is denoted as *dropping factor* φ_0_. Then the relationship φ = φ(h) can be described with an exponential function:

φ(h) = φ_0_ − e^[(0.85 − h) − 1.0]^. (9)

For the investigated PTFE dropping factor was calculated as φ_0_ = 0.40% ± 0.73% (U95%) that shows a small dielectric dispersion of PTFE’s permittivity and corresponds to the requirements set for the material of protective coverage/coating. In comparison, even the most light-weight foams from the investigated ones PUR foams LAST-A-FOAM^®^ FR-3703, density ρ ≈ 48 kg/m^3^, porosity η_g_ = 1 − ρ/ρ_p_ = 96% and ε_t_ = 1.08 (1 kHz), exhibit nearly two times higher dropping factor φ_0_ ≈ 0.7%, where ρ_p_ = 1280 kg/m^3^ is density of monolithic polyurethane. Dropping factor, calculated from the approximated permittivity spectra of PUR foams, is given in dependence of PUR foams’ density in Table 1 and Figure 9.

### 2.6. Permittivity Spectra

**Figure 6:**
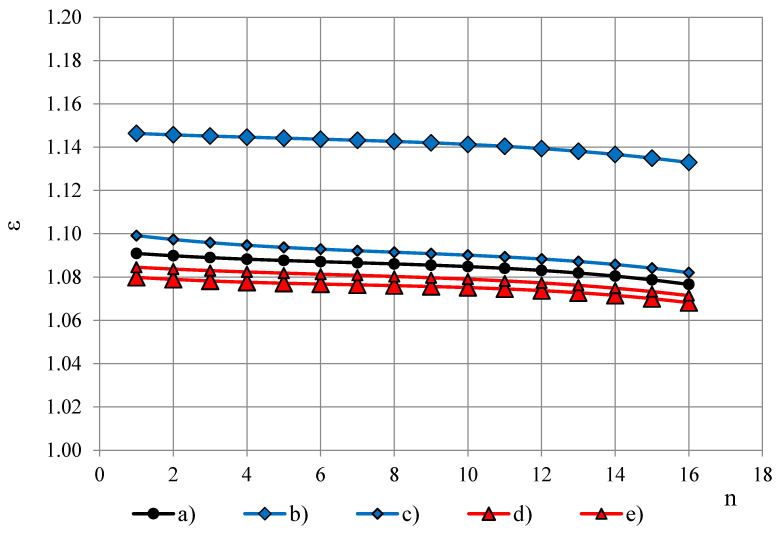
Permittivity spectra of PUR foams LAST-A-FOAM^®^ FR-3703, ρ = 48 kg/m^3^: (a) ε_t_, (b) ε_T_ at PTFE film 0.20 mm, (c) ε_T_ at PTFE film 0.04 mm, (d) ε_T′_ at PTFE film 0.20 mm and (e) ε_T′_ at PTFE film 0.04 mm.

**Figure 7:**
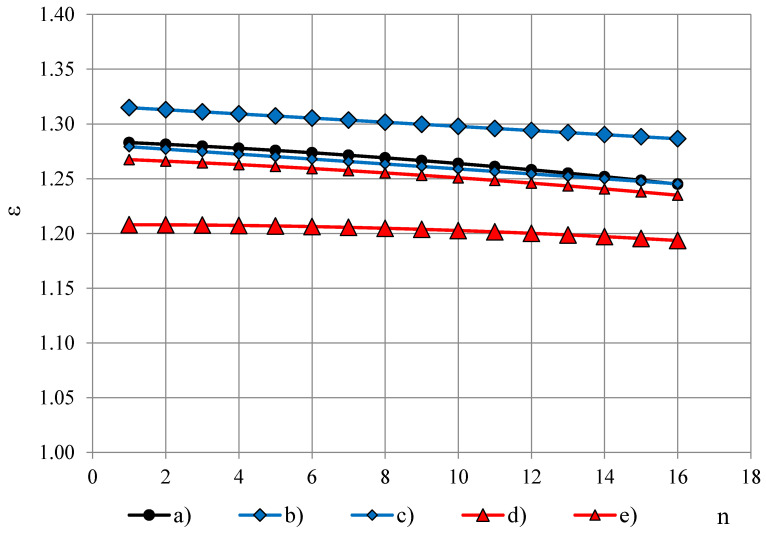
Permittivity spectra of PUR foams Sika-150, ρ = 144 kg/m^3^: (a) ε_t_, (b) ε_T_ at PTFE film 0.20 mm, (c) ε_T_ at PTFE film 0.04 mm, (d) ε_T′_ at PTFE film 0.20 mm and (e) ε_T′_ at PTFE film 0.04 mm.

**Figure 8:**
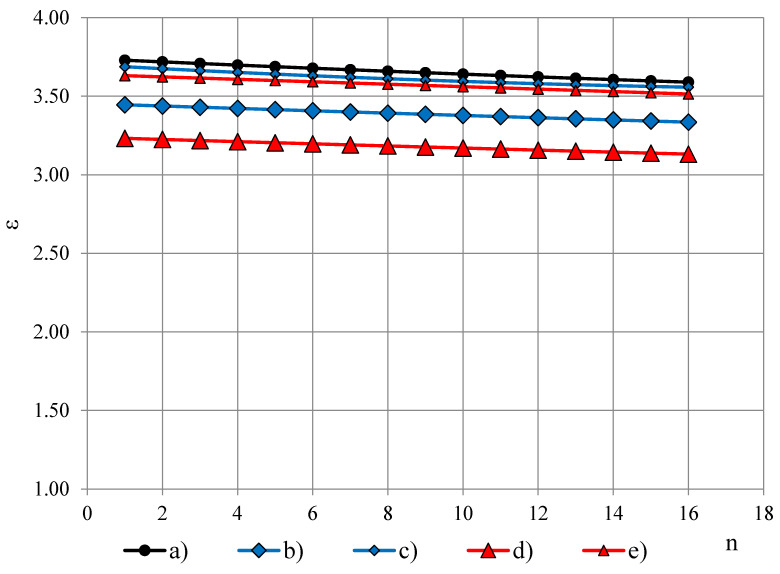
Permittivity spectra of lab-made monolithic polyurethane, ρ_p_ = 1280 kg/m^3^: (a) ε_t_, (b) ε_T_ at PTFE film 0.20 mm, (c) ε_T_ at PTFE film 0.04 mm, (d) ε_T′_ at PTFE film 0.20 mm and (e) ε_T′_ at PTFE film 0.04 mm.

The true permittivity spectra ε_t_ of (a) light-weight PUR foams LAST-A-FOAM^®^ FR-3703, ρ = 48 kg/m^3^, (b) PUR foams Sika-150, ρ = 144 kg/m^3^ and (c) monolithic lab-made polyurethane, density 1280 kg/m^3^ are given in Figure 6, Figure 7 and Figure 8 together with permittivity spectra ε_T_ and ε_T′_. The permittivity spectra are approximated with the third order polynomials of frequency ordeal number n.

Experimental data of the true permittivity ε_t_ is summarized in Table 1, at 1 kHz, where U95%—the expanded uncertainty. The ε_t_ values of the same density PUR foams are similar for the four groups that correlate with results in [5].

**Figure 9:**
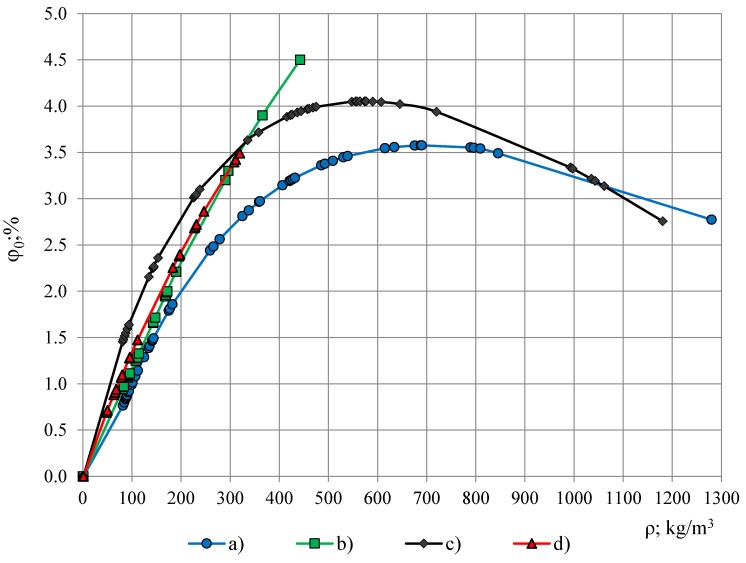
Dropping factor in dependence of PUR foams’ density: (a) lab-made foams, (b) lab-made biofoams, (c) Sika JSC foams and (d) General Plastics Manufacturing Company foams.

Table 1 gives data of dropping factor φ_0_, calculated from the approximated permittivity spectra. Figure 9 depicts φ_0_ in dependence of density of (a) lab-made PUR foams, (b) lab-made PUR biofoams, (c) Sika JSC PUR foams and (d) General Plastics Manufacturing Company PUR foams. For the light-weight PUR foams the dependence φ_0_(ρ) is nearly linear, φ_0_ ≤ 1.5%, up to densities 100 kg/m^3^ for all four groups of PUR foams. The highest dropping factor is exhibited by Sika JSC PUR foams and the lab-made PUR biofoams at densities 550–650 kg/m^3^: φ_0_ ≈ 4.0–4.5%. After the maximum the value of φ_0_ decreases to the dropping factor value of monolithic polyurethane. All the values of PUR foams’ dropping factor are 10–40 times higher than dropping of PTFE films 0.02/0.04 mm: 0.09% and 0.12%, which facilitates small modifications of permittivity spectra caused by a PTFE film on the OSA sensor’s active area.

The measured values of permittivity ε_T_ and ε_T′_ are also summarized in Table 1, at 1 kHz. Values of ε_T_ characterise the overall impact of the PTFE film on the true value of PUR foams’ permittivity. Values of ε_T′_ give the measured permittivity value of PUR foams’ when coating with a PTFE film of OSA sensor’s active area is simulated and calibration has to be made with a PTFE film.

### 2.7. Estimation of Modifications

To estimate impact of the PTFE films on the PUR foams’ true permittivity spectra ε_t_(f_n_), *the average modification factor* η_Taver_ is defined for permittivity spectra ε_T_(f_n_) and ε_T′_(f_n_):

Δε_tT_(f_n_) = [ε_t_(f_n_) − ε_T_(f_n_)]/ε_t_(f_n_), Δε_tT′_(f_n_) = [ε_t_(f_n_) − ε_T′_(f_n_)]/ε_t_(f_n_), n = 1, 2, …, 16;

(10) $$\eta_{\mathrm{Taver}}=[\sum_{1}^{16}\Delta \varepsilon_{\mathrm{tT}}\left(f_{n}\right)]/16,\eta_{T\prime \mathrm{aver}}=[\sum_{1}^{16}\Delta \varepsilon_{\mathrm{tT}\prime }\left(f_{n}\right)]/16.$$

When the entire measured permittivity spectrum ε_T_(f_n_) or ε_T′_(f_n_) lies above the true permittivity spectrum ε_t_(f_n_), η_Taver_ < 0.0; when it lies below ε_t_(f_n_), η_Taver_ > 0.0. If spectrum ε_T_(f_n_) or ε_T′_(f_n_) intersects the spectrum ε_t_(f_n_), absolute values of differences |Δε_tT_(f_n_)| and |Δε_tT′_(f_n_)| have to be taken in Equation (10).

The spectra ε_t_(f_n_) and ε_T′_(f_n_) are evaluated towards a criterion of small modifications as well:

|ε_t_(f_n_) − ε_T_(f_n_)| ≤ Δε_t_, |ε_t_(f_n_) − ε_T′_(f_n_)| ≤ Δε_t_, n = 1, 2, ..., 16;

(11) $$\Delta \varepsilon_{t}=\eta_{0}\varepsilon_{\mathrm{taver}}=\eta_{0}[\sum_{1}^{16}\varepsilon_{t}\left(f_{n}\right)]/16;$$

where η_0_ is a small modification parameter. Modification of the true permittivity spectrum ε_t_ by presence of a PTFE film is considered as small when the measured values of permittivity ε_T_ or ε_T′_ at each frequency f_n_ differ from the true permittivity values ε_t_ no more than by a pre-defined value ±Δε_t_, calculated as a η_0_ fraction from the average value ε_taver_ of the true permittivity spectrum values ε_tn_:

ε_t_(f_n_) − η_0_ ε_taver_ ≤ ε_T_(f_n_) ≤ ε_t_(f_n_) + η_0_ ε_taver_; ε_t_(f_n_) − η_0_ ε_taver_ ≤ ε_T′_(f_n_) ≤ ε_t_(f_n_) + η_0_ ε_taver_. (12)

i.e., the entire spectrum ε_T_(f_n_) or ε_T′_(f_n_) has to be situated inside a zone around the true permittivity spectrum ε_t_(f_n_) defined by Equation (12). The value of η_0_ depends on the required accuracy of measurements. 

The impact of PTFE films on the PUR foams’ true permittivity spectra ε_t_(f_n_) was estimated for PUR foams of Table 1, according to the following methodology. First, the numerical value of average modification factor was calculated for ε_T_(f_n_) and ε_T′_(f_n_). Depending on the required accuracy, a certain limit value for the average modification factor is set. As an example, η_lim_ was set to 3.5%, the calculated η_Taver and_ η_T′aver_ values were compared to η_lim_ and conclusions made. Then the average permittivity value of the true permittivity spectra ε_taver_ was calculated, the small modification parameter was set to values η_0_ = 1, 2, ..., 5% and satisfaction of the criterion of small modifications by the spectra ε_T_(f_n_) and ε_T′_(f_n_) was tested. A PC code was compiled for numerical calculations.

## 3. Results

### 3.1. Average Modification Factor

Numerical calculations of the average modification factor η_Taver_ for permittivity spectra ε_T_(f_n_), corresponding to the coverage with a PTFE film, thickness 0.20 mm, gave the following results, Table 1: (1) Lab-made PUR foams: −4.0% ≤ η_Taver_ ≤ 7.3% at PUR foams’ density 84–1280 kg/m^3^; (2) lab-made PUR biofoams: −4.3% ≤ η_Taver_ ≤ 1.6% at 84–442 kg/m^3^; (3) Sika JSC PUR foams: −4.9% ≤ η_Taver_ ≤ 7.1% at 85–1181 kg/m^3^ and (4) Gen. Plast. PUR foams −5.2% ≤ η_Taver_ ≤ −0.7% at 85–320 kg/m^3^.

Numerical calculations of η_T′aver_ for permittivity spectra ε_T′_(f_n_), corresponding to simulating coating with a PTFE film, thickness 0.20 mm, gave the following results, Table 1: (1) lab-made PUR foams η_T’aver_ = 2.8–14.0% at 84–1280 kg/m^3^, (2) lab-made PUR biofoams η_T’aver_ = 3.1–9.5% at 84–442 kg/m^3^, (3) Sika JSC PUR foams η_T’aver_ = 2.6–13.6% at 85–1181 kg/m^3^ and (4) Gen. Plast. PUR foams η_T’aver_ = 0.9–5.2% at 48–320 kg/m^3^. It can be concluded that the average modifications caused by the PTFE film of thickness 0.20 mm are quite high in both cases: up to 10–14% and exceed η_lim_ = 3.5%.

For permittivity spectra ε_T_(f_n_), corresponding to the coverage with a PTFE film, thickness 0.04 mm, the following numerical values of η_Taver_ were acquired, Table 1: (1) Lab-made PUR foams η_Taver_ = −0.4–1.2% at 84–1280 kg/m^3^, (2) lab-made PUR biofoams η_Taver_ = −0.5–0.4% at 84–442 kg/m^3^, (3) Sika JSC PUR foams η_Taver_ = −0.5–1.3% at 85–1181 kg/m^3^ and (4) Gen. Plast. PUR foams η_Taver_ = −0.6–0.4% at 48–320 kg/m^3^. It can be concluded that the |η_Taver_| remains less than 1.3% as well as less than η_lim_ = 3.5% in density range of PUR foams 84–1280 kg/m^3^, which might be acceptable for certain practical applications, depending on the required accuracy, e.g., to perform a quick evaluation of rigid PUR foams’ permittivity in field conditions (Thermal insulation, shielding structures, etc.). Calibration of the spectrometer can be made in air, but the permittivity measurements of the rigid PUR foams’ test object—with a PTFE film temporarily covered underneath the test object, on the OSA sensor’s active area, to protect the OSA sensor’s electrodes from the adverse side effects caused by the PUR foams’ test object. Thus, an operational flexibility can be retained, since the OSA sensor does not have to be permanently coated with the PTFE film.

**Figure 10:**
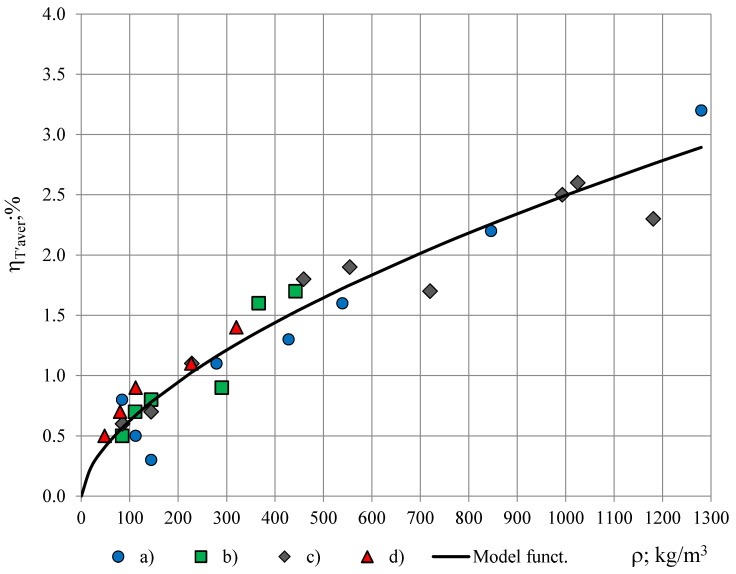
Average modification factor η_T′aver_ in dependence of PUR foams’ density: (a) Lab-made petrochemical, (b) lab-made biofoams, (c) Sika JSC and (d) General Plastics Manufacturing Company.

For permittivity spectra ε_T′_(f_n_), corresponding to simulating coating with a PTFE film, thickness 0.04 mm, the dependence of η_T′aver_ on PUR foams’ density, Table 1, is depicted in Figure 10.

The relationship η_T′aver_ = η_T′aver_(ρ) follows a similar trend for all four groups of PUR foams and can be described with a function:

η_T′aver_(ρ) = Kρ^0.60^, where K = 0.04. (13)

It can be concluded that with a PTFE film, thickness 0.04 mm, the average modification factor of the true permittivity spectra remains less than η_lim_ = 3.5% for density range of rigid PUR foams 48–1280 kg/m^3^. For the light- to medium-weight PUR foams of density ρ ≤ 220 kg/m^3^, applied in shielding structures, building, heat and cold insulation etc. [23,24,25,26], the average modification factor is even smaller: η_T′aver_ ≤ 1.0%. At similar densities the values of η_T′aver_ are similar for the four investigated groups of PUR foams: (a) lab-made petrochemical, (b) lab-made biofoams, (c) Sika JSC and (d) General Plastics Manufacturing Company. It can be concluded that the modifications, caused by a simulated permanent coating of a PTFE film, thickness 0.04 mm, on the active area of the OSA sensor, might be acceptable for certain practical applications, depending on the required accuracy.

### 3.2. Small Modification Criterion

Numerical calculations, made for the lab-made and Sika JSC PUR foams showed that for a temporary coverage with a PTFE film, thickness 0.20 mm, the criterion of small modifications is satisfied by permittivity spectra ε_T_(f_n_) for small values of η_0_ = 1.0%, 1.5% and 2.0% only in a narrow density range 250–550 kg/m^3^ and, thus, represent no practical interest.

When PTFE film is of thickness 0.04 mm, the criterion is satisfied by permittivity spectra ε_T_(f_n_) of the lab-made PUR foams when η_0_ = 1.0% in a density range 84–550 kg/m^3^ and when η_0_ = 1.5–5.0% in a density range 84–1280 kg/m^3^. The trend is similar for lab-made PUR biofoams, Sika JSC and Gen. Plast. PUR foams, too. Such modifications might be acceptable for certain practical applications, depending on the required accuracy.

Numerical calculations, made for the lab-made and Sika JSC PUR foams, showed that in the simulation of a permanent PTFE film’s coating, thickness 0.20 mm, the criterion is satisfied by permittivity spectra ε_T′_(f_n_) only starting from η_0_ = 3.0%, up to densities ρ ≈ 100 kg/m^3^, at η_0_ = 4.0%, up to ρ_f_ ≈ 250 kg/m^3^ and at η_0_ = 5.0%, up to ρ_f_ ≈ 350 kg/m^3^. At the small values η_0_ = 1.0%, 1.5% and 2.0% the criterion is not satisfied by the spectra ε_T′_(f_n_) in density range 85 kg/m^3^–1280 kg/m^3^. The trend is similar for lab-made PUR biofoams and Gen. Plast. PUR foams. The modifications caused by the PTFE film of thickness 0.20 mm are quite high and likely unacceptable for practical applications.

When the PTFE film is of thickness 0.04 mm, the criterion is satisfied by permittivity spectra ε_T′_(f_n_) of the lab-made PUR foams at: (a) η_0_ = 1.0%, up to densities ρ ≈ 200 kg/m^3^, (b) η_0_ = 1.5%, up to ρ_f_ ≈ 430 kg/m^3^, (c) η_0_ = 2.0%, up to ρ_f_ ≈ 700 kg/m^3^, (d) η_0_ = 3.0%, up to ρ ≈ 1 100 kg/m^3^ and (e) η_0_ = 4.0 and 5%, up to ρ ≈ 1280 kg/m^3^. The trend is similar for lab-made PUR biofoams, Sika JSC and Gen. Plast. PUR foams. The modifications might be acceptable for certain practical applications, depending on the required accuracy.

## 4. Evaluation of Measurement Uncertainties

### 4.1. Measurement Uncertainties Due to Air Gaps

Measurement uncertainties due to air gaps (gaps) between active surface of the OSA sensor and bottom of the cylindrical samples (PUR foams and PTFE) are evaluated. The gaps are formed by cavities in the bottom surface of a sample from one side and active surface of the sensor, from the other. The active surface of the sensor was evaluated and assumed to be ideally smooth. The cavities in the bottom surface of the cylindrical samples are caused mainly by (1) foaming defects—spherical/ellipsoidal segments of gaseous macro-bubbles and (2) surface-smoothing, etc., processing. Samples with macro-segments of diameter > 3 mm, significantly exceeding the average diameter of the cells were excluded from experiments. To reduce processing cavities, the samples were cut from the central part of parallelepipeds, having area of the bottom surfaces at least two times as large as the area of a sample’s bottom.

Gaps between bottom surface of the sample and active surface of the sensor were investigated with steel indicators, thickness 0.03–0.50 mm. The sample was placed on the sensor and indicators of thickness 0.50, 0.40, ..., 0.10 mm and 0.09, 0.08, ..., 0.03 mm were slid around the perimeter of the contact surface. When the indicator entered the gap without resistance, it was assumed that its height is equal to the thickness of indicator. An indicator of the least thickness 0.03 mm was applied to determine width and depth of a gap.

It was identified that PUR foams’ samples of densities 40–200 kg/m^3^ each had 1–3 cavities of height h = 0.03–0.06 mm, width w = 7–22 mm and depth d = 1–3 mm. PUR foams’ samples, ρ_f_ > 200 kg/m^3^ and PTFE samples have only comparatively small cavities of h < 0.03 mm and w < 7 mm that are not taken into account further. All cavities are situated along the perimeter of sample’ bottom surface.

**Figure 11:**
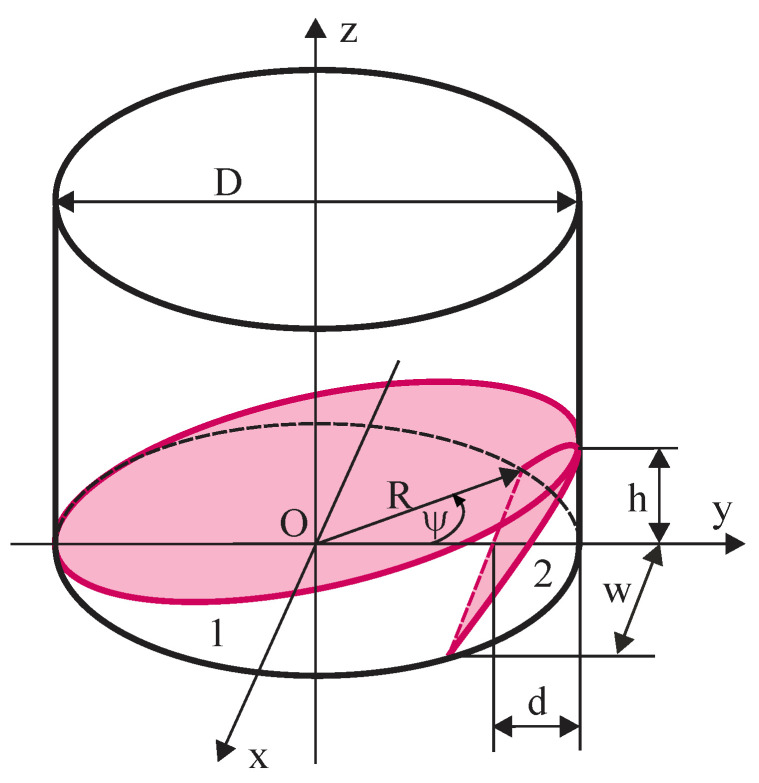
Gaps between the bottom surface of a sample and active area of the sensor: 1) a penetrating gap and 2) a perimetral gap.

Gaps are assumed to have shape of a cylindrical wedge, Figure 11, where w = 2a = $2\sqrt{(w\left(2R-d\right)}$, b = *R*(1 − cos*ψ*), *ψ*—polar angle and R—radius of the sample’s bottom. Volume of the wedge:

(14) $$V=\frac{hR^{2}}{3}\left(\frac{3sin\psi -3\psi cos\psi -sin^{3}\psi }{1-cos\psi }\right),0\leq \psi \leq 2\pi .$$

Two types of wedge-shaped gaps are considered: perimetral (*ψ* ≪ π/2) and penetrating (*ψ* = π). A penetrating gap is formed when a sample is put with one end of its bottom diameter on a PTFE spacer of thickness h_s_, while the other end is built on the perimeter of the sensor, in a point-contact. Volume of a penetrating gap at h_s_ = 1.0 mm equals V_0_ = 795.2 mm^3^.

Table 2 gives numerical values of volumes V of the perimetral gaps and of proportion K = *V*/*V*_0_. Modelling the limit case when each sample of density below 200 kg/m^3^ has three largest perimetral cavities of dimensions h = 0.06 mm, w = 22 mm and d = 2.9 mm, k is calculated:

(15) $$K=\sum_{1}^{3}V_{0}/V_{i}=257.$$

To estimate influence of a penetrating air gap (ε_air_ ≈ 1.00059) on the measured value of true permittivity ε_t_, a spacer—a PTFE strip of thickness h_s_ = 1.0 mm and width 2 mm was placed on the perimeter of the sensor at depth ~ 2 mm. A sample was built on the spacer and the sensor, forming a penetrating gap. The true permittivity ε_t_ and the measured value of the true permittivity ε_tpn_ with a penetrating gap was measured for PUR foams’ samples, ρ_f_ = 48 and 144 kg/m^3^, Table 3. Three measurements were made for each sample. The change in the value of the true permittivity due to a penetrating gap is calculated as:

Δε_tpn_ = ε_t_ − ε_tpn_. (16)

The change Δε_tpn_ due to gaps is assumed to be proportional to the total volume and location of gaps. Even with the maximum amount of the biggest perimetral gaps their total volume is ≈ 260 times less than the volume V_0_ of a penetrating gap of height 1 mm, Equation (15). A penetrating gap is situated above the whole active surface of the sensor, therefore Δε_tpn_ characterises an averaged influence of location. On the contrary, the experimentally identified gaps are perimetral gaps, situated along the perimeter of sensor’s active surface, where intensity of the excitation field is the weakest. It is concluded that for PUR foams, ρ_f_ < 200 kg/m^3^, the upper boundary of the change in the value of the true permittivity caused by the identified perimetral gaps is Δε^U^_tpm_ = Δε_tpn_/k, Table 4. A sample with no cavities provides the lower boundary: Δε^L^_tpm_ = 0.0.

It is expected that all the values of ε_t_ for a PUR foams’ sample, ρ_f_ < 200 kg/m^3^, with perimetral cavities, fall in the mentioned limits, therefore:

U95%^g^ = 1/3(Δε^U^_tpm_ − Δε^L^_tpm_); (17)

where U95%^g^ is the expanded uncertainty of measurement, based on a standard uncertainty multiplied by a coverage factor k = 2, which for a normal distribution corresponds to a coverage probability of approximately 95% [27,28].

### 4.2. Measurements Uncertainty of ε_t_

The measurement uncertainty of true permittivity ε_t_ is evaluated for lab-made PUR foams, ρ_f_ = 50, 112, 144, 427, 846 kg/m^3^, monolithic polyurethane 1280 kg/m^3^ and PTFE according to ISO Guide to the Expression of Uncertainty in Measurement (GUM) [27,28]. The accuracy of the dielectric spectrometer in conditions of reproducibility was evaluated with expanded uncertainty U95%^S^ = ± 0.01. In Type A evaluation series of n = 3–4 statistically independent, non-destructive observations are made for each sample of PUR foams and monolithic polyurethane; for the PTFE sample n = 18. Measurement uncertainty arises mainly due to inhomogeneous density of the samples, air gaps between the sample and active surface of the sensor etc. Since the expanded uncertainty of true permittivity due to perimetral gaps U95%^g^ is several orders smaller than U95%^S^ due to the limited accuracy of the spectrometer (Table 4), the air gaps are not taken into account. Estimate of the input quantity and sensitivity coefficient are calculated: $\bar{\varepsilon_{t}}=\frac{1}{n}\overset{n}{\underset{1}{\sum }}\varepsilon_{\mathrm{ti}}$ and c_1_ = 1.0. Attributing normal distribution to the measurand, effective degrees of freedom *ν*_eff_ of the combined standard uncertainty u_c_(ε_t_) associated with the output estimate are estimated from the Welch—Satterthwaite formula:

(18) $$\nu_{\mathrm{eff}}=u_{c}{}^{4}\left(\varepsilon_{t}\right)/\sum_{i=1}^{N}[u_{i}^{4}\left(\varepsilon_{t}\right)/\nu_{i}],\nu_{i}=n-1\mathrm{and}N=1,$$

where N is the number of the input quantities. The numerical results for standard uncertainty u(ε_t_) for Type A evaluation at frequencies f_1_, f_7_ and f_16_ are given in Table 5.

The measurement uncertainty due to limited accuracy of dielectric spectrometer and digital calliper is considered in Type B evaluation. Measurements of sample’s thickness were made with a digital calliper of limited resolution δh and accuracy δδh. However, taking into account the high allowance for thickness: 2.0–2.5 times larger than the penetration depth, δh and δδh practically have no influence on the measured value of ε_t_ and are not taken into account. That yields the standard uncertainty u(ε_t_) = U95%^S^/2 = ± 0.05.

The combined standard uncertainty is calculated from Type A and Type B uncertainty budget $u_{c}\left(\varepsilon_{t}\right)=\sqrt{\sum_{1}^{N}c_{i}^{2}u^{2}\left(x_{i}\right)}$, where N = 2. When a number of distributions of whatever form are combined it can be shown that, apart from in exceptional cases, the resulting probability distribution tends to the normal form in accordance with the Central Limit Theorem [28]. Then for a normal distribution the expanded uncertainty is calculated as U95% = ku_c_(ε_t_), where k = 2 is the coverage factor.

For PUR foams, ρ_f_ = 427 kg/m^3^ and f_7_ = 640 Hz, the measurement result for a series is ε_t_ = 1.865 ± 0.011. The reported expanded uncertainty U95% of ε_t_ measurement is stated as the standard uncertainty of measurement multiplied by the coverage factor k = 2, which for a normal distribution corresponds to a coverage probability of approximately 95%.

Due to technological processes at foaming the heavier PUR foams are more inhomogeneous, especially lab-made ones. That increases the standard uncertainty u(ε_t_) in Type A type evaluation, characterising the repeatability. For the most of the PUR foams input of Type A uncertainties is smaller or comparable to the input of Type B uncertainties. No significant difference in values of u_c_(ε_t_) and U95% was identified for true permittivity spectra as measured and the approximated ones. It is concluded that approximation smooths out fluctuations in experimental data, retaining the dominating trend.

### 4.3. Measurement Uncertainty of Dropping Factor

The measurement uncertainty of dropping factor φ_0_ is evaluated for lab-made PUR foams, ρ_f_ = 50, 112, 144, 427, 846 kg/m^3^, monolithic polyurethane 1280 kg/m^3^ and PTFE according to GUM. In Type A type evaluation series of n = 3–4 statistically independent, non-destructive observations are made for each sample of PUR foams and monolithic polyurethane; for the PTFE sample n = 5. The permittivity spectra are approximated with the 3rd-order polynomials for PUR foams and with the 2nd-order polynomials for PTFE. The model function:

φ_0_ = [ε(f_3_) − ε(f_14_)]/ε(f_3_) = 1 − ε(f_14_)/ε(f_3_), (19)

where ε(f_3_) and ε(f_14_)—input quantities. Estimates of ε_3_ = ε(f_3_) and ε_14_ = ε(f_14_) and corresponding sensitivity coefficients are calculated:

(20) $$\bar{\varepsilon_{3}}=\frac{1}{n}\sum_{1}^{n}\varepsilon_{3i}\;\mathrm{and}\;\bar{\varepsilon_{14}}=\frac{1}{n}\sum_{1}^{n}\varepsilon_{14i};$$

(21) $$c_{1}=\frac{\partial \varphi }{\partial \varepsilon_{3}}=\varepsilon_{14}/{\left(\varepsilon_{3}\right)}^{2}\;\mathrm{and}\;c_{2}=\frac{\partial \varphi }{\partial \varepsilon_{14}}=-1/\varepsilon_{3}.$$

The measurement uncertainty due to limited accuracy of dielectric spectrometer is considered in Type B evaluation that yields the standard uncertainty u(ε_3_) = u(ε_14_) = U95%^S^/2 = ± 0.05. Uncertainty contribution of input quantities is expressed as u_1_(φ) = c_1_u(ε_3_) and u_2_(φ) = c_2_u(ε_14_) and the combined standard uncertainty is calculated from Type A and Type B uncertainty budget. Then for a normal distribution the expanded uncertainty U95% = ku_c_(φ_0_), where k = 2.

For PUR foams, ρ_f_ = 427 kg/m^3^ the measurement result for a series is φ_0_ = 0.037 ± 0.011. The reported expanded uncertainty U95% of ε_t_ measurement is stated as the standard uncertainty of measurement multiplied by the coverage factor k = 2, which for a normal distribution corresponds to a coverage probability of approximately 95%.

For PTFE the measurement result for a series is φ_0_ = 0.40% ± 0.68%, Table 6. Uncertainties larger than measured values are common in measurements where the measurand value is expected to be zero or close to it, as the dropping factor of PTFE.

No significant difference in values of u_c_(φ_0_) and U95% was identified for dropping factor φ_0_ calculated from the permittivity spectra as measured and the approximated ones.

### 4.4. Measurement Uncertainty of Complex Samples

The Type A measurement uncertainty of the measured permittivity spectra ε_T_ and ε′_T_ is evaluated for lab-made PUR foams, ρ_f_ = 50, 112, 144, 427, 846 kg/m^3^ and monolithic polyurethane 1280 kg/m^3^ according to GUM. In Type A type evaluation series of n = 3–4 statistically independent, non-destructive observations are made for each PUR foams sample and monolithic polyurethane, Table 7. Measurement uncertainty arises mainly due to inhomogeneous density and structure of the samples, air gaps between the sample, the PTFE films and active surface of the sensor, etc.

Experimental investigation with the indicators showed no significant difference in the dimensions of the gaps in the case of the PTFE films of thickness 0.04 mm in comparison to single samples. With PTFE film of thickness 0.20 mm the gaps between the sample, the PTFE films and active surface of the sensor were around two times higher and wider than in case of single samples. Since the same spectrometer is used, the uncertainties of ε_T_ and ε′_T_ can be expected to be of the same order as those of ε_t_. Additional experimental and theoretical research is necessary to evaluate the uncertainties of the measured permittivity spectra ε_T_ and ε′_T_ more precisely.

## 5. Discussion

The impact of PTFE films, thickness 0.20 mm and 0.04 mm, in covering and simulated coating of the active area of OSA capacitive sensor’ electrodes on the true dielectric permittivity spectra of rigid petrochemical- and bio-origin PUR foams is estimated by means of (1) a modification factor and (2) a small modification criterion. Although the quantitative results for estimation of impact of PTFE films are valid for the given OSA sensor with its topography of electric field, the developed methodology can be applied for OSA capacitive sensors of other configuration and dimensions of electrodes, independent of the sensor’s particular technical solution.

The methodology can be applied for solving similar problems in dielectric permittivity measurements of other cellular plastics: polyvinyl chloride, polyethylene, polystyrene, polypropylene, polyolefin, etc., foams as well as for impact estimation of other plastic films, e.g., polyethylene. For practical realisation of the PTFE protective coating, further investigations are needed on the relevant technologies, sustainability of the coating against mechanical rupture, wear and de-lamination during exploitation, etc.

The PTFE films’ protection can be useful in dielectric permittivity measurements of rigid PUR foams’ samples as well as in the non-destructive evaluation of PUR foams’ industrial items.

## Figures and Tables

**Table 1:** Data of the permittivity spectra ε_t_, ε_T_ and ε_T′_.

N	Materials	Single Samples				PTFE Film Coverage				PTFE Film Coating (Simul.)			
						0.20 mm		0.04 mm		0.20 mm		0.04 mm	
		ρ;kg/m^3^	η_g_;%	ε_t_ ± U95%	φ_0_;%	ε_T_	η_Taver_;%	ε_T_	η_Taver_;%	ε_T′_	η_T′aver_;%	ε_T′_	η_T′aver_;%
(1) PTFE													
1	PTFE; h = 14.37 mm	2 158	0	2.10 ± 0.01	0.4	2.08	0.8	2.09	0.3	1.96	6.5	2.07	1.4
(2) Lab-made petrochemical PUR foams													
1	PUR foams	84	93	1.14 ± 0.011	0.8	1.19	−4.0	1.15	−0.4	1.11	2.8	1.13	0.8
2	PUR foams	112	91	1.20 ± 0.010	1.2	1.23	−3.1	1.20	−0.6	1.16	3.1	1.19	0.5
3	PUR foams	144	89	1.26 ± 0.011	1.4	1.31	−3.7	1.27	−0.9	1.22	3.5	1.26	0.4
4	PUR foams	279	78	1.50 ± 0.013	2.6	1.55	−2.1	1.50	0.1	1.42	5.5	1.49	1.1
5	PUR foams	427	67	1.84 ± 0.010	3.2	1.83	0.5	1.83	0.4	1.71	6.6	1.81	1.3
6	PUR foams	539	58	2.12 ± 0.021	3.5	2.09	1.7	2.11	0.5	1.91	10.1	2.09	1.6
7	PUR foams	846	34	2.61 ± 0.019	3.5	2.48	5.0	2.58	1.2	2.33	11.0	2.56	2.2
8	Monol. PUR	1280	0	3.64 ± 0.015	2.8	3.37	7.3	3.59	1.2	3.17	14.0	3.55	3.2
(3) Lab-made PUR biofoams													
1	PUR biofoams	84	93	1.13 ± 0.014	1.1	1.18	−4.3	1.13	−0.5	1.09	3.1	1.12	0.5
2	PUR biofoams	111	91	1.22 ± 0.012	1.3	1.26	−3.2	1.22	−0.4	1.17	3.8	1.21	0.7
3	PUR biofoams	144	89	1.23 ± 0.010	1.7	1.27	−3.1	1.23	−0.2	1.17	4.7	1.22	0.8
4	PUR biofoams	290	77	1.61 ± 0.016	3.2	1.60	0.4	1.60	0.3	1.48	7.3	1.58	0.9
5	PUR biofoams	366	71	1.74 ± 0.021	3.9	1.73	0.7	1.73	0.5	1.62	6.5	1.71	1.6
6	PUR biofoams	442	65	1.95 ± 0.027	4.5	1.93	1.6	1.96	0.4	1.78	9.5	1.93	1.7
(4) Sika JSC, petrochemical PUR foams													
1	Sika-80	85	93	1.14 ± 0.011	1.5	1.20	−4.9	1.15	−0.5	1.11	2.6	1.13	0.6
2	Sika-150	144	88	1.26 ± 0.011	2.2	1.31	−3.7	1.27	−1.0	1.23	2.8	1.25	0.6
3	Sika-240	228	81	1.42 ± 0.010	3.0	1.45	−2.2	1.42	−0.1	1.36	3.9	1.40	1.1
4	Sika-450	459	61	1.95 ± 0.014	4.0	1.92	1.5	1.93	1.1	1.80	7.8	1.91	1.8
5	Sika-600	554	53	2.09 ± 0.015	4.1	2.05	1.9	2.08	0.8	1.93	7.7	2.05	1.9
6	Sika-700	720	39	2.59 ± 0.018	3.9	2.49	3.8	2.55	1.6	2.34	9.8	2.55	1.7
7	Sika-930	993	16	3.13 ± 0.019	3.3	2.98	4.9	3.11	1.0	2.84	9.3	3.06	2.5
8	Sika-1000	1 025	13	3.26 ± 0.021	3.3	3.06	5.9	3.21	1.2	2.88	11.3	3.17	2.6
9	Monol. PUR M-960	1 181	0	3.78 ± 0.013	2.8	3.51	7.1	3.73	1.3	3.26	13.6	3.70	2.4
(5) General Plastics Manufacturing Company , petrochemical PUR foams													
1	FR-3703	48	96	1.08 ± 0.011	0.7	1.14	−5.2	1.09	−0.6	1.08	0.9	1.08	0.5
2	FR-4305	80	94	1.14 ± 0.010	1.2	1.20	−4.4	1.15	−0.4	1.12	1.7	1.14	0.7
3	FR-3707	112	92	1.20 ± 0.012	1.5	1.24	−3.6	1.20	−0.2	1.17	2.3	1.19	0.9
4	FR-4315	227	83	1.42 ± 0.010	2.7	1.45	−2.1	1.42	0.1	1.37	3.9	1.41	1.1
5	FR-7120	320	76	1.60 ± 0.011	3.5	1.61	−0.7	1.59	0.4	1.51	5.2	1.58	1.4

**Table 2:** Parameters of perimetral gaps.

N	φ; rad	w = 2a; mm	d; mm	V; mm^3^		K = V/V_0_	
				h = 0.03 mm	h = 0.06 mm	h = 0.03 mm	h = 0.06 mm
1	0.1787	8	0.4	0.0230	0.0460	0.00003	0.00006
2	0.2241	10	0.6	0.0452	0.0903	0.00006	0.00011
3	0.2699	12	0.8	0.0786	0.1573	0.00010	0.00020
4	0.3164	14	1.1	0.1260	0.2519	0.00016	0.00032
5	0.3635	16	1.5	0.1900	0.3800	0.00024	0.00048
6	0.4115	18	1.9	0.2738	0.5477	0.00034	0.00069
7	0.4606	20	2.3	0.3809	0.7619	0.00048	0.00096
8	0.5108	22	2.9	0.5152	1.0305	0.00065	0.00130

**Table 3:** Influence of a penetrating air gap on the true permittivity, 1 kHz.

N	ρ_f_; kg/m^3^	ε_t_			ε_tpn_			Δε_tpn_		
		f_1_	f_7_	f_16_	f_1_	f_7_	f_16_	f_1_	f_7_	f_16_
1	48	1.09	1.09	1.08	1.07	1.07	1.06	0.02	0.02	0.02
2	144	1.27	1.25	1.23	1.19	1.18	1.16	0.08	0.07	0.07

**Table 4:** Boundaries of changes caused by perimetral gaps, 1 kHz.

N	Density; kg/m^3^	Δε^L^_tpm_			Δε^U^_tpm_			U95%^g^		
		f_1_	f_7_	f_16_	f_1_	f_7_	f_16_	f_1_	f_7_	f_16_
1	48	0.0	0.0	0.0	0.00008	0.00008	0.00008	0.00003	0.00003	0.00003
2	144	0.0	0.0	0.0	0.00031	0.00027	0.00027	0.00010	0.00009	0.00009

**Table 5:** Measurement uncertainties of the true permittivity spectra ε_t_.

N	Density; kg/m^3^	u(ε_t_); Type A			u_c_(ε_t_)			U95%		
		f_1_	f_7_	f_16_	f_1_	f_7_	f_16_	f_1_	f_7_	f_16_
	PUR foams									
1	50	0.0015	0.0000	0.0003	0.0052	0.0050	0.0050	0.010	0.010	0.010
2	112	0.0015	0.0002	0.0012	0.0052	0.0050	0.0051	0.010	0.010	0.010
3	144	0.0030	0.0016	0.0008	0.0058	0.0052	0.0051	0.012	0.010	0.010
4	427	0.0027	0.0019	0.0007	0.0057	0.0053	0.0051	0.011	0.011	0.010
5	846	0.0190	0.0081	0.0044	0.0196	0.0095	0.0067	0.039	0.019	0.013
6	1280	0.0083	0.0057	0.0062	0.0097	0.0076	0.0080	0.019	0.015	0.016
	PTFE									
1	2177	0.0023	0.0021	0.0020	0.0055	0.0054	0.0054	0.011	0.011	0.011

**Table 6:** Measurement uncertainties of dropping factor φ_0_.

N	Density; kg/m^3^	u(φ_0_); Type A	u_c_(φ_0_)	U95%
	PUR foams			
1	50	0.0039	0.0076	0.015
2	112	0.0015	0.0064	0.013
3	144	0.0015	0.0062	0.012
4	427	0.0010	0.0054	0.011
5	846	0.0055	0.0068	0.014
6	1280	0.0020	0.0027	0.005
	PTFE			
7	2177	0.0014	0.0034	0.0068

**Table 7:** Measurement uncertainties of the measured permittivity spectra (at f_7_, permittivity spectra as measured).

N	Density; kg/m^3^	u(ε_T_); Type A		u(ε′_T_); Type A	
		0.20 mm	0.04 mm	0.20 mm	0.04 mm
1	50	0.0014	0.0010	0.0005	0.0007
2	112	0.0120	0.0006	0.0055	0.0006
3	144	0.0012	0.0013	0.0104	0.0006
4	427	0.0014	0.0016	0.0175	0.0005
5	846	0.0033	0.0174	0.0139	0.0009
6	1280	0.0205	0.0017	0.0037	0.0022

## Data Availability

Data is contained within this article.
